# The Prevalence of Vitamin D Insufficiency and Deficiency and Their Relationship with Bone Mineral Density and Fracture Risk in Adults Receiving Long-Term Home Parenteral Nutrition

**DOI:** 10.3390/nu9050481

**Published:** 2017-05-10

**Authors:** Navaporn Napartivaumnuay, Leah Gramlich

**Affiliations:** 1 Department of Medicine, University of Alberta, Edmonton, AB T6G 2R3, Canada; lg3@ualberta.ca; 2 Nutrition Services, Alberta Health Services, Edmonton, AB T5J 3E4, Canada

**Keywords:** home parenteral nutrition, vitamin D, vitamin D insufficiency, vitamin D deficiency, bone mineral density, fracture

## Abstract

It has been demonstrated that low bone mass and vitamin D deficiency occur in adult patients receiving home parenteral nutrition (HPN). The aim of this study is to determine the prevalence of vitamin D insufficiency and deficiency and its relationship with bone mineral density (BMD) and fracture risk in long-term HPN patients. *Methods*: A retrospective chart review of all 186 patients in the HPN registry followed by the Northern Alberta Home Parenteral Nutrition Program receiving HPN therapy >6 months with a 25 (OH) D level and BMD reported were studied. *Results*: The mean age at the initiation of HPN was 53.8 (20–79) years and 23 (37%) were male. The mean HPN duration was 56 (6–323) months and the most common diagnosis was short bowel syndrome. Based on a total of 186 patients, 62 patients were categorized based on serum vitamin D status as follows: 1 (24.2%) sufficient, 31 (50%) insufficient and 16 (25.8%) deficient. Despite an average of 1891 IU/day orally and 181 IU/day intravenously vitamin D, the mean vitamin D level was 25.6 ng/mL (insufficiency) and 26.2 ± 11.9 ng/mL in patients with the highest 10-year fracture risk. *Conclusion*: Suboptimal vitamin D levels are common among patients on long-term HPN despite nutrient intake that should meet requirements.

## 1. Background

Osteoporosis contributes to morbidity in patients with intestinal failure on long-term home parenteral nutrition (HPN). The factors that contribute to this are multi-factorial. The role of vitamin D sufficiency and its relation to bone disease has been studied [1] in this population.

Patients requiring HPN have intestinal failure, most commonly secondary to short bowel syndrome (SBS), which results in poor nutrient absorption, particularly fat malabsorption, which may play a role in vitamin D deficiency [2]. Lack of adequate ultraviolet exposure from sunlight due to chronic illness and medications that affect vitamin D metabolism are additional risk factors found in HPN patients [3]. Vitamin D deficiency is defined as 25 (OH) D below 20 ng/mL (50 nmol/L), and vitamin D insufficiency as a 25 (OH) D of 21–29 ng/mL (52.5–72.5 nmol/L) [4]. Vitamin D levels are inversely associated with parathyroid hormone levels until they reach 30 to 40 ng/mL. Moreover, intestinal calcium transport increased by 45 to 65% in women when vitamin D levels were increased from an average of 20 to 32 ng/mL. Given this, a vitamin D level of 30 ng/mL or greater can be considered to indicate sufficient vitamin D [5].

Metabolic bone disease (MBD) in patients on long-term HPN can be asymptomatic or associated with bone pain or fractures which may occur as a result of minimal or no trauma. The pathogenesis of MBD is related to several factors which may be HPN-related and/or related to the patients’ underlying disease and general lifestyle. Parenteral nutrition factors contributing to osteoporosis include calcium, vitamin D, phosphate deficiencies and amino acid infusion. High doses of amino acid infusion, particularly exceeding 2 g/kg/day increases urinary calcium excretion [6]. Amino acids have been shown to cause hypercalciuria by increasing renal blood flow and hence glomerular filtration rate. The primary disease associated with intestinal failure may cause malabsorption of calcium and vitamin D. Chronic inflammation and medications, in particular corticosteroids, have a negative impact on bone health.

The prevalence of osteoporosis in HPN patients with intestinal failure (IF) has been found to be 67% and is dependent on body mass index and age when IF occurred [7]. Multiple studies have demonstrated that the risk of fractures increases with declining bone mineral density (BMD) in patients who are not on HPN [8,9].

In the Northern Alberta HPN program, oral vitamin D supplementation (7500 IU/day) is prescribed for 8 weeks for patients when vitamin D levels are <20 ng/mL (<50 nmol/L) and reduced to 2000 IU/day once vitamin D is at a sufficient level. If patients fail to achieve optimal vitamin D level with oral vitamin D supplements, intramuscular vitamin D is given. If osteoporosis was diagnosed, parenteral pamidronate 30–60 mg, adjusted according to renal function is given every 3 months. Routine assessment of serum hydroxyvitamin D (25 (OH) D), (considered the best measurement for assessing vitamin D status) and routine BMD measurement has become standard practice in our program [10,11].

The purpose of this study is to determine the prevalence of vitamin D insufficiency and deficiency and its relationship with BMD and 10-year fracture risk in adult patients requiring long-term HPN.

## 2. Materials and Methods

### 2.1. Patients

Data from the Northern Alberta HPN program within the Canadian HPN Registry was aggregated and analyzed. Data on patients’ demographic including age, sex, body mass index (BMI), HPN indication, duration on HPN, HPN regimen, medications (steroids, bisphosphonates, anticoagulant, oral vitamin D and calcium supplements), vitamin D status and BMD were extracted.

All patients routinely received one standard parenteral multivitamin (without vitamin K) which contained 200 IU of vitamin D_3_ (cholecalciferol) per day. This is less than the Osteoporosis Canada recommendation of 400–2000 IU/day for all adult year round [12].

### 2.2. Patient Groups

Eligible patients were categorized by mean serum vitamin D status as follows: (1) vitamin D sufficient, 25 (OH) D >30 ng/mL (>75 nmol/L); (2) insufficient, 25 (OH) D 21–29 ng/mL (50–75 nmol/L); (3) deficient, 25 (OH) D <20 ng/mL (<50 nmol/L). The method used to detect 25 (OH) D was high-performance liquid chromatography (HPLC).

The presence and the degree of metabolic bone disease was assessed based on BMD measurement at the lumbar spine and femoral neck using a dual-energy X-ray absorptiometry (DEXA) scan. *T*-score (the number of standard deviations from the mean BMD value of young sex-matched subject) was used to define the diagnostic categories in adults age ≥50 years as follows: normal, a *T*-score within 1 SD; reduced (osteopenia), a *T*-score below −1 SD but less than −2.5 standard devaiation (SD); severely reduced (osteoporosis), a *T*-score equal or below −2.5 SD and severe osteoporosis is low BMD with fragility fracture noted. The BMD *Z*-score (number of standard deviations from normal values corrected for sex and age) was used to describe the presence and degree of bone disease in patients age <50 years as follows: normal, a *Z*-score within 1 SD and reduced, a *Z*-score below −1 SD.

### 2.3. Inclusion and Exclusion Criteria

All adult patients followed by the Northern Alberta Home Parenteral Nutrition Program between 2002 and 2014 who required HPN for >6 months were included. Patients provided informed consent for participation in the HPN registry which is approved by the Human Ethics Research Board at University of Alberta (Pro00053988). Ethics approval was obtained for this study. Patients with underlying malignancy were excluded from the study as were patients whom serum vitamin D levels and BMD were not recorded in the HPN Registry.

### 2.4. Outcome Measurement

The primary outcome studied was the prevalence of vitamin D sufficiency, insufficiency and deficiency in long-term HPN patients from the Northern Alberta HPN Program. Secondary outcomes studied included correlation of vitamin D status with bone mineral density and 10-year fracture risk in patients on long-term HPN.

### 2.5. Study Design

This is a retrospective observational cohort study.

### 2.6. Statistical Analysis

Data are presented as mean ± standard deviation, median, number (%) and range (in parentheses). For each patient, mean vitamin D levels during HPN are reported. Bone mineral density at least 6 months after starting HPN were used for evaluation. All analyses were performed using SPSS (version 20, IBM Corp, Armonk, NY, USA). A *p*-value of less than 0.05 was used to determine statistical significant.

## 3. Results

In total, 62 of 186 patients receiving long-term HPN with measured serum vitamin D level and BMD were identified and included into the analysis. The mean age at start of HPN was 53.8 (20–79) years and 23 (37%) were male. Mean HPN duration was 56 (6–323) months, and the most common non-malignant cause of intestinal failure was short bowel syndrome (61.3%). Other indications included surgical complications (enterocutaneous fistula 16%, motility disorder 11.3% and mucosal defect 6.4%). Table 1 presents a summary of demographic and clinical variables including sex, age at start of HPN, BMI, indication for HPN, duration of HPN, HPN regimen and medications (steroids, bisphosphonates, anticoagulant, calcium, and oral vitamin D supplementation) used during the study period.

The mean vitamin D level was 25.6 ng/mL (8.11–55.6 ng/mL) and 62 patients were categorized into sufficient 15 (24.2), insufficient 31 (50), and deficient 16 (25.8) based on the serum vitamin D levels.

Individual HPN patients had between 1 and 34 measurements of vitamin D levels done during their HPN therapy. The mean vitamin D level and vitamin D status are presented in Table 2.

Out of 62 patients, 64.5% had abnormal bone mineral density and the majority of these patients were over 50 years of age. Eighty-two percent of patients with osteoporosis received treatment with parenteral bisphosphonates every 3 months and had an annual follow up of BMD. Fifty percent of our 62 patients had a moderate to high 10-year fracture risk (Table 3).

Among the patients studied, four (6.5%) had history of fragility fractures. When comparing the patients with fragility fracture to those without fracture, the BMD of all patients with fragility fracture was significantly lower than the no fracture group (*p* < 0.001). The HPN duration and oral vitamin D supplement dosage were not statically significantly different amongst those with fragility fracture compared to those without fracture. However, the vitamin D level was significantly higher in those patients with fragility fractures, 35.5 ± 9.45 ng/mL (88.78 ± 23.63 nmol/L) compared to those 24.92 ± 9.12 ng/mL (62.3 ± 22.8 nmol/L) without fracture group (*p* = 0.029). All patients with fragility fracture had received parenteral bisphosphonate infusion.

HPN patients age <50 years old had similar vitamin D levels in the normal BMD group compared to the abnormal BMD group (*p* = 0.45). In HPN patients >50 years old, the vitamin D level was higher in those with osteopenia and osteoporosis compared to the normal BMD group but this was not statistically significant (*p* = 0.31). The mean vitamin D levels were considered suboptimal in all groups and were the lowest in the low fracture risk group, 23.9 ± 9.92 ng/mL (59.73 ± 24.80 nmol/L). Those patients with highest fracture risk did not have a lower mean vitamin D level than those with lowest fracture risk. The ten-year fracture risk did not correlate with vitamin D level (Table 4).

## 4. Discussion

Vitamin D insufficiency and deficiency is prevalent in patients receiving long-term HPN [7,13,14,15,16]. Previous studies had shown similar results of vitamin D status in their HPN patients (Table 5). The mean duration of HPN in the population we studied is longer than other studies at 56 (6–323) months. The mean vitamin D level is also higher than most studies at 25.60 ng/mL (8.11–55.6 ng/mL) but is still in the insufficient vitamin D range. In previous studies, approximately 50% or more of their HPN patients had vitamin D deficiency while only 25% of our study population had vitamin D deficiency. Higher doses of oral vitamin D seems to result in a higher vitamin D level. Comparable for all studies was the standard parenteral multi in their HPN, which contains 200 IU of vitamin. Similarly, a study done by Ellegard et al. did not find any association between vitamin D level and BMD [13].

Since suboptimal levels of vitamin D are found at a high rate in long-term HPN patients despite high doses of vitamin D supplementation, initial and subsequent follow up of vitamin D levels are necessary. The European Society of Parenteral Nutrition on chronic intestinal failure guideline 2016 recommended monitoring vitamins and trace elements at six-month intervals and investigating for metabolic bone disease annually [17].

To evaluate osteopenia and osteoporosis in our HPN patients, we chose to analyze the T-scores in patients age >50 years old and Z-scores in patients age <50 years old from the BMD measurement. From our study, we found that 64.5% of our HPN patients have low BMD which is similar to many studies. One study by Raman et al. [18], a Canadian perspective on HPN and metabolic bone disease (MBD), found a MBD prevalence of 76% and MBD correlated with the duration of HPN. Another study showed that the prevalence of MBD in HPN patients ranged between 40% and 100% [19,20]. When compared to the general population worldwide, approximately 10% of women aged 60 years and 20% by the age of 70 years would suffer from osteoporosis [21]. This demonstrates that HPN patients have a higher prevalence of metabolic bone disease compared to the general population.

Patients with fragility fracture had the worst BMD score but had higher levels of vitamin D throughout their participation in the HPN program (*p* = 0.029) while on lower doses of oral vitamin D. This emphasizes that vitamin D alone is not the only cause of poor bone quality and fracture risk. Rather, multiple factors are likely playing a role in these patients with chronic intestinal failure. This may, in part, explain why our data did not reveal any significant association between vitamin D status and BMD results.

In the 16 HPN patients with high 10-year fracture risk, we found out that the average age was 56 years old (29–70), 69% had short bowel syndrome, 88% were female, 19% were underweight (BMI < 18.5 kg/m^2^), 88% smoked and the average time on HPN was 64 months.

Other secondary causes of osteoporosis (hypogonadism, hyperthyroidism, hyperparathyroidism) need to be investigated and appropriately treated in order to effectively treat osteoporosis [22]. Other medications including subcutaneous teriparatide could be considered in patients refractory to bisphosphonate therapy who develop severe osteoporosis without hyperparathyroidism, end stage renal disease or osteosarcoma risk [23]. Other vitamins such as vitamin K in HPN patients have been investigated through the Canadian HPN Registry. HPN patients receiving vitamin K supplementation had significantly higher BMDs than patients not receiving vitamin K in their HPN solution. Therefore, vitamin K supplementation in the HPN regimen might be of benefit especially for patients with very little oral intake and no contraindication to vitamin K [24]. In our study, we did not routinely give vitamin K supplement because the majority of our HPN patients continue oral intake. We assume that there is some production of vitamin K from bacterial production and another source of vitamin K is the parenteral lipid emulsion. About 16% of our patients are on anticogulation therapy and vitamin K supplementation is contraindicated.

A limitation of this study which may preclude the demonstration of a positive correlation between vitamin D status and BMD was the evaluation of only a single BMD result which may not be an adequate representation of the patient’s bone health. The difference in vitamin D levels in our study compared to others may be due to the different methodologies for vitamin D measurement. For instance, our study used HPLC while other studies used radioimmunoassay assessment [7,14] and some did not mention what method was used [15,16].

## 5. Conclusions

It is important to monitor vitamin D levels at least at six-month intervals [17], and adequately supplement with appropriate vitamin D route, such as intramuscular or separate vitamin D parenteral infusion and higher dosages given that the prevalence of vitamin D insufficiency and deficiency in HPN patients is common. In patients on long-term HPN, BMD should be monitored annually and osteoporosis should be treated [17]. Further studies need to be undertaken to establish the appropriate method of delivery, dose and duration of oral vitamin D supplementation in intestinal failure patients. Factors such as vitamin K supplementation should also be taken into consideration as it may have potential benefit to BMD.

## Figures and Tables

**Table 1:** Demographic and clinical characteristics of patients receiving long-term home parenteral nutrition (HPN).

Number of HPN Patients	62
Gender Male, No of patients (%)	23 (37)
Mean ageat start of HPN, years (range)	53.8 (20–79)
Mean BW, kg	59.6 (39.5–97.7)
Mean BMI, kg/m^2^ (range)	22 (15.4–32.5)
Indications for HPN, No. (%)	
Short bowel syndrome	38 (61.30)
- Crohn’s disease	20 (52.63)
- Bowel ischemia	11 (28.94)
- Bowel atresia	1 (2.63)
- Other	6 (15.79)
Mucosal defect	4 (6.40)
Motility disorder	7 (11.30)
Surgical Complications	
- Enterocutaneous fistula	10 (16)
Others	3 (4.8)
HPN Duration and Regimen	
Mean duration HPN, months (range)	56 (6–323)
Mean days per week, days (range)	6.32 (2–7)
Mean kcal/kg/day	30.7 (22–47.6)
Protein g/kg/day	1.3 (0.25–2.26)
Dextrose g/kg/day	3.43 (0.35–6.37)
lipid g/kg/day	0.6 (0.07–1)
Medications, No. of Patient (%)	
Vitamin D	36 (58)
Calcium	20 (32.25)
Anticoagulant	10 (16.12)
Steroid	4 (6.4)
Bisphosphonate	37 (59.67)
Mean Dose of oral vitamin D supplement, IU/day (range)	1891 (0–7000)
Mean Dose of IV vitamin D, IU/day (range)	181 (57–200)
Mean dose of oral calcium supplement, mg (range)	1167.5 (0–2000)

BW: body weight; BMI: body mass index.

**Table 2:** Mean vitamin D level and status in 62 HPN patients.

Vitamin D Level, ng/mL (Range)	
Total, Mean	25.6 (8.11–55.6)
Vitamin D status, No. (%)	
Sufficient (>30 ng/mL)	15 (24.2)
Insufficiency (20–30 ng/mL)	31 (50)
Deficiency (<20 ng/mL)	16 (25.8)

**Table 3:** Bone mineral density and 10-year fracture risk in 62 HPN patients.

Bone Mineral Density (BMD)	No. (%)
Normal BMD	22 (35.5)
Low BMD	40 (64.5)
- age <50	7 (17.5)
- age >50	33 (82.5)
- osteopenia	16 (40)
- osteoporosis	17 (42.5)
Osteoporosis with bisphosphonate treatment	14 (82.3)
10 years Fracture risk	No. (%)
Low	4 (6.45)
Average	25 (40.32)
Moderate	16 (25.80)
High	16 (25.80)
Unknown	1 (1.61)

**Table 4:** Ten-year fracture risk and its association with vitamin D level in 62 HPN patients.

Ten Years Fracture Risk	*n* = 62	Vitamin D level, ng/mL (Mean ± SD)	
Low	4	23.89 ± 9.92
Average	25	25.20 ± 8.38
Moderate	16	26.47 ± 9.11
High	16	26.02 ± 11.90
unknown	1	18.8 ± 0

SD: stardard devitation.

**Table 5:** Vitamin D level, BMD and vitamin D dosage of HPN patients in several studies.

Intestinal Failure with TPN	*n*	Duration (mo)	Mean Vitamin D (ng/mL)	Vitamin D Deficiency <20 ng/mL (%)	Low BMD (%)	Parenteral Vitamin D (IU)	Oral Vitamin D (IU)	Vitamin D Levels and BMD Correlation
Thomson et al. 2011 (Manitoba) [14]	22	33.5 (1–177)	16.8 ± 0.4	68		166 ± 43.9	79.5 ± 102	-
Ellegard et al. 2012 (Sweden) [13]	106 ***		17.2 ± 10.8 then 25.6 ± 10	67	88 (44, 44)	160 ± 40	200–800 *	None
Kumar et al. 2012 (S. Alberta) [15]	14 (5)	36.4	20.8 (17.2–21.2)	55 **			5000 (4000–7143)	-
Bharadwaj et al. 2014 (Cleveland) [16]	79	39.2	24.5 ± 12.7	44.3		200	7143–21,428	-

Three patients use individualized doses of vitamin D; ** vitamin D 11–30 ng/mL; *** only 35 patients on HPN. Only one study by Ellegard et al. studied the correlation between vitamin D level and BMD.
